# Northern Sydney Local Health District (NSLHD) eating disorders project

**DOI:** 10.1186/2050-2974-2-S1-O26

**Published:** 2014-11-24

**Authors:** Rochelle Wildman, Andrea Taylor, Michael Paton

**Affiliations:** 1 Mental Health Drug and Alcohol, Northern Sydney Local Health District, Sydney, Australia

## 

NSLHD identified the need to improve the care of people with eating disorders, following clinical reports of increasing number and complexity of eating disorders presentations. A Clinical Redesign Project was subsequently established in February 2013.

Inpatient data trends were analysed and demonstrated there had been an increase of approximately 80 admissions and 800 bed days per annum for eating disorders since 2010/11.

To further understand this clinical trend, a file audit was conducted of all admitted patients with a diagnosis of an eating disorder during 2012/13. There were 124 presentations, consisting of 85 individual patients, 90% of whom were female. Anorexia Nervosa accounted for 43% of episodes and 59% of bed days, Bulimia Nervosa for 24% of episodes and 8% of bed days, and Eating Disorder Not Otherwise Specified (EDNOS) for 32% of episodes and 33% of bed days.

Key issues identified during stakeholder consultations will be presented as will the proposed model of care, to be implemented over the next 3-5 years, subject to available funding.

This project has been successful in collecting and analysing clinical data relating to patients, processes, and staff involved in the care of patients with eating disorders in NSLHD. We have been able to design and prioritise solutions to the issues and build stakeholder support, which will be integral for the successful implementation of the model of care.

This abstract was presented in the **Service Initiatives** stream of the 2014 ANZAED Conference.

